# Usefulness of low iodine diet in managing patients with differentiated thyroid cancer - initial results

**DOI:** 10.2478/v10019-011-0017-4

**Published:** 2011-06-24

**Authors:** Margareta Dobrenic, Drazen Huic, Marijan Zuvic, Darko Grosev, Ratimir Petrovic, Tatjana Samardzic

**Affiliations:** Department of Nuclear Medicine and Radiation Protection, University Hospital Center Zagreb, Zagreb, Croatia

**Keywords:** low iodine diet, urine iodine concentration, differentiated thyroid cancer, radioiodine

## Abstract

**Background:**

Low iodine diet (LID) is recommended in patients with differentiated thyroid cancer before radioiodine administration. Patients with increased thyroglobulin (Tg) level, but negative ^131^I whole body scan present diagnostic and therapeutic dilemma. This study was designed to evaluate the benefit of a two-week LID in patients with elevated serum Tg levels and negative ^131^I whole body scans.

**Patients and methods.:**

For the impact assessment of two-week LID on radioiodine tissue avidity, radioiodine scans before and after LID were compared. Sixteen patients with serum Tg > 2 μg/L, negative Tg-antibodies, and negative radioiodine scans underwent two-week LID before the ^131^I administration. Fourteen patients underwent diagnostic scanning and two patients received radioiodine therapy. Iodine concentration in the morning urine specimens were measured in each patient, a day before and 15^th^ day after starting LID.

**Results:**

Following self-managed LID, patients were able to significantly reduce their iodine body content by 50% (range 28–65%, p<0,001). 13 patients (82%) accomplished mild iodine deficiency (50-99 μg/L) and one patient (6%) achieved targeted moderate iodine deficient state (<50 μg/L). All diagnostic post-LID scans were negative. Both post-therapy ^131^I scans showed radioiodine accumulation outside of normal ^131^I distribution (neck region and diffuse hepatic uptake). This study demonstrated that two-week LID is effective way to decrease total body iodine content, although without a visible effect on post-LID diagnostic ^131^I scans.

**Conclusions:**

A more stringent dietary protocol and longer iodine restriction period are probably needed to achieve targeted moderate iodine deficiency in patients preparing for ^131^I administration. This might result in higher radioiodine avidity of thyroid remnant/metastases.

## Introduction

A follow-up of patients with differentiated thyroid cancer (DTC) is a lifelong process. The goals of monitoring after the initial therapy are to maintain the adequate thyroid hormone therapy and to detect persistent or recurrent thyroid cancer. When used at the same time, serum thyroglobulin (Tg) levels measurement and ^131^I whole body scanning offer best possibilities in the patients’ follow-up.^1^ Serum Tg is a tumour marker and radioiodine scans localize tumour sites. To enhance ^131^I uptake in whole body scans, thyrotropin (TSH) levels should be above 30 mU/L prior to ^131^I administration for either diagnostic or therapeutic purposes.^2^ This could be achieved by a withdrawal of thyroid hormone therapy, resulting in symptomatic hypothyroidism before and during the testing period. Alternative to the thyroid hormone withdrawal is an administration of recombinant human TSH (rh-TSH) in euthyroid patients.^3^

When ^131^I scanning or therapy is planed, patients are also instructed to follow a low iodine diet (LID), which basically means avoiding the iodine-rich food and iodine-containing medications.^4^ LID is designed to decrease the total body stable iodine concentration prior to radioiodine administration. Pluijmen *et al.* demonstrated the increase of radioiodine uptake by 65% in thyroid remnant and also longer effective half-life of ^131^I, both of which contributed to the increase of the absorbed radiation dose.^5^ Most centres advise a two-week low iodine diet prior to the ^131^I administration.^4^,^6^

Urinary iodine excretion is a good marker of the recent dietary iodine intake. According to the World Health Organization’s report, profile of iodine concentrations in the morning urine specimens provides an adequate assessment of the recent dietary iodine intake.^7^ Furthermore, according to the same report, urinary iodine concentration less than 50 μg/L reflects a moderate iodine deficiency, while urinary iodine concentration >50 μg/L and <99 μg/L indicates a mild iodine deficient state.

Patients with elevated serum Tg level and negative ^131^I whole body scan present a diagnostic and a therapeutic challenge. One of the possibilities for such a finding is that neoplastic thyroid tissue is still capable to produce thyroglobulin but is unable to accumulate ^131^I, suggesting tumour dedifferentiation.^8^ The other possibility is that thyroid cancer metastases/remnant tissue is blocked with stable iodine. The identification of the Tg production site may dictate appropriate treatment modalities, for instance, surgery in the case of lymph node metastases and resectable distant lesions, or ^131^I therapy in radioiodine-avid metastases and thyroid remnant tissue.

The aim of this study was to evaluate the influence of decreased total body iodine level on radioiodine avidity of possible thyroid remnants, tumour recurrences or metastases after a two-week LID.

## Methods

### Patients

Sixteen patients (11 women, 5 men, median age 55) with DTC who fulfilled the criteria of serum Tg concentration above 2 μg/L, negative thyroglobulin-antibodies (TgAt<20 U/mL) and negative ^131^I whole body scan were selected for this study. All patients underwent total thyroidectomy and ^131^I thyroid remnant ablation as an initial therapy. Ten patients (63%) had papillary and 6 patients (37%) had follicular cancer. A postsurgical ^131^I administration was performed in all patients in hypothyroid state with 888-5550 MBq (24–150 mCi) 4–6 weeks after the surgery. Applied ^131^I activity depended on tumour size and initial cancer extension. In the follow-up all patients had negative ^131^I whole body scans, but in seven patients the other diagnostic studies (computed tomography, chest x-ray and fine needle aspiration (FNA) cytology) suggested recurrent thyroid cancer. In five of them small nodules in lungs were found and in one patient bone lesions were suspected. One patient had lymph node metastasis in the neck region (proven by FNA cytology) but was not willing to the surgical treatment. Trying to find out whether there is any uptake of radioiodine, the same patients were scheduled for control ^131^I whole body scans 12–18 months after the last scanning, but now after two-week LID. All of them were withdrawn from L-thyroxin therapy for four weeks prior to ^131^I scanning. The serum Tg concentration was measured with elevated TSH (TSH>30 mU/L in all patients).

All fourteen patients who received diagnostic activity of ^131^I had serum Tg level between 2 and 6 μg/L before LID. Since the empiric ^131^I therapy is justified in patients with Tg levels > 10 μg/L and negative ^131^I whole body scan^9^, two of our patients underwent the empiric radioiodine therapy (serum Tg value was 22.4 μg/L in one patient and 24.9 μg/L in another). In those patients gradually rising serum Tg levels were observed after the initial treatment. There are not any data about receiving iodinated contrast media in the six months prior to the diagnostic or therapeutic ^131^I administration. Epidemiological and clinical characteristics of the selected patients are summarized in Table 1.

### Low iodine diet

Low iodine diet was explained to the patients and they were sent home with a list of dietary recommendations (Table 2). Patients have been told to follow LID for two weeks prior to the ^131^I administration.

### Urine iodine

Two morning urine specimens were obtained from each patient to assure the adequate diet preparation has been achieved. The first urine sample was taken a day before starting a LID and the second sample after a two-week LID performance, a few hours before the ^131^I application. Both specimens were immediately deep frozen for further determinations of urinary stable iodine. The determination of urinary iodine concentration was based on the manual spectrophotometric measurement of Sandell-Kolthoff reduction reaction catalysed by iodine.^10^–^12^ Spectrophotometer Camspect M350 Double Beam was used. Sensitivity of the method is 5 μg of iodine/L. WHO recommends the expression of urinary iodine concentration as a simple iodine concentration (μg/L), without the urinary creatinine measurement or 24 h urine collection.^7^ Final results were expressed as iodine concentration (μg/L).

### Dietary efficacy

Categories of iodine depletion were based upon WHO’s criteria of the iodine nutrition status.^7^ Urinary iodine concentration less than 50 μg/L reflects a moderate iodine deficient state and was the aimed value in this study. Urinary iodine concentration >50 μg/L and <99 μg/L indicates a mild iodine deficient status and is suboptimal, but still an adequate preparation for the radioiodine administration. Urinary iodine concentration >100 μg/L and < 199 μg/L represents the adequate iodine nutrition status.

### Radioiodine (^131^I) scintigraphy

Gamma camera imaging of ^131^I distribution in patients was performed using either DIACAM (Siemens Gammasonics, Inc.,Hoffman Estates, IL) single-head camera or SYMBIA E (Siemens Gammasonics, Inc.,Hoffman Estates, IL) dual-head camera. Both cameras have similar characteristics: rectangular field of view (53.3×38.7 cm), 9.5 mm NaI (Tl) detector crystal thickness and high-energy collimators. Patient scintigraphy acquisitions included: a whole body planar anterior scan (acquisition matrix: 256 × 1024), and static anterior scan of the neck and the thorax region with a preset number of 150 000 counts.

At the same day of the ^131^I administration, the morning urine sample for the urinary iodine concentration measurement was taken. Fourteen patients who underwent diagnostic post-LID radioiodine scans received 185 MBq (5 mCi) of ^131^I activity each, and acquisitions were performed 48h after the radioiodine administration. Two patients with Tg level higher than 22 μg/L have been scheduled for radioiodine therapy and received 3700 MBq (100 mCi) and 7400 MBq (200 mCi) of ^131^I, respectively. Post-therapy scans following LID were made 72 h after the radioiodine administration.

Radioiodine scans were assessed visually.

### Statistical analysis

T-test: paired two sample for means was used to test the difference between groups. A statistical result was considered significant if p< 0.001.

## Results

### Dietary performance

**FIGURE 1:**
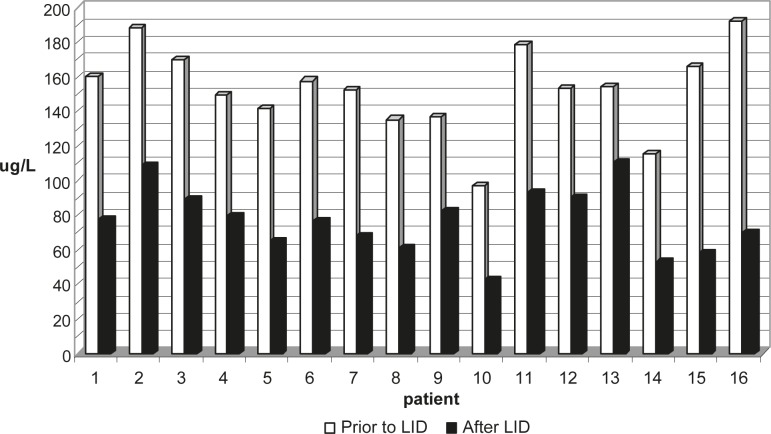
Urine iodine concentration (μg/L) for each patient prior to and after low iodine diet (LID).

Urine iodine concentration values based on the single morning urine sample are presented in Figure 1 and Table 3. Fifteen patients (94%) had an adequate iodine intake prior to LID and, therefore were optimally iodine nourished. One patient (6%) was mild iodine deficient before LID. Following self-managed LID, patients were able to significantly reduce their iodine body content by 50% (mean±SD: 50±9, range: 28–65, p<0.001). 88% of patients were iodine deficient after two-week LID. 82% of patients (n=13) accomplished mild iodine deficiency (50-99 μg/L) and one patient (6%) achieved a targeted moderate iodine deficient state (<50 μg/L). Two patients (12%) had the iodine sufficient status (>100 μg/L) even after two-week low iodine diet preparation.

### Post–low iodine diet (post-LID) radioiodine scans

**FIGURE 2:**
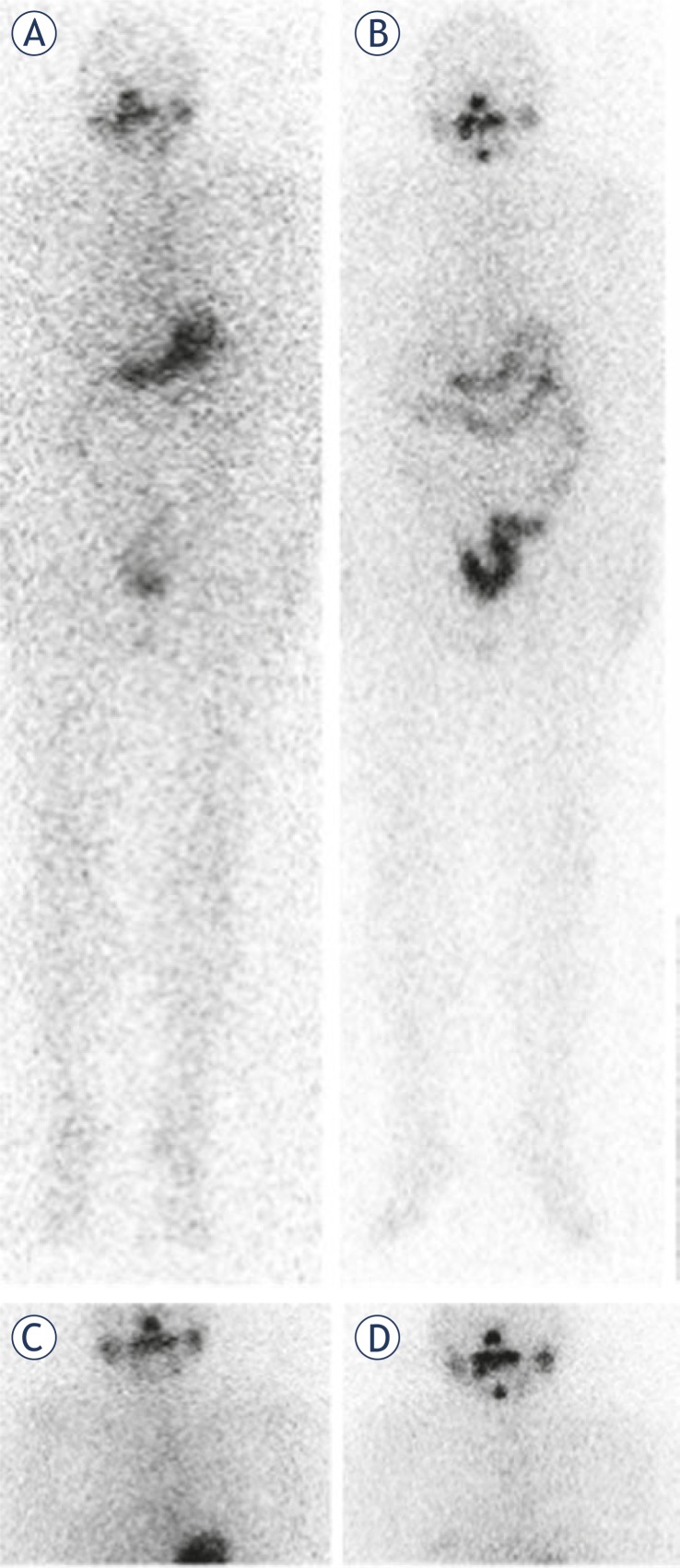
Radioiodine scans of the patient who received 3700 MBq (100 mCi) of ^131^I. The ^131^I uptake is visible in the neck region. Prior to a low iodine diet (LID), the patient had urinary iodine concentration of 97.1 μg/L, and post-LID value was 42.5 μg/L. Anterior whole body scans (A) prior to LID and (B) post LID; static scans of the neck and the thorax region (C) prior to LID and (D) post LID.

Fourteen patients underwent diagnostic ^131^I scan after two-week LID and all post-LID radioiodine scans were negative. Six of those patients had morphological findings suggestive of recurrent thyroid cancer in neck lymph node, lungs and bones. Patients with lung and bone metastases were mild iodine deficient after LID. The patient with a neck lymph node metastasis was sufficient iodine nourished even after two-week LID performance. On the other hand, on both post-therapy ^131^I scans some radioiodine accumulation outside of the normal ^131^I distribution was visible. The patient who received 3700 MBq of ^131^I had focal radioiodine accumulation in the neck region. He was the only one who had the aimed moderate iodine deficient status after LID and, therefore, was optimally prepared for the ^131^I administration. His serum Tg value prior to LID and the empiric ^131^I therapy was 22.4 μg/L (Figure 2).

**FIGURE 3:**
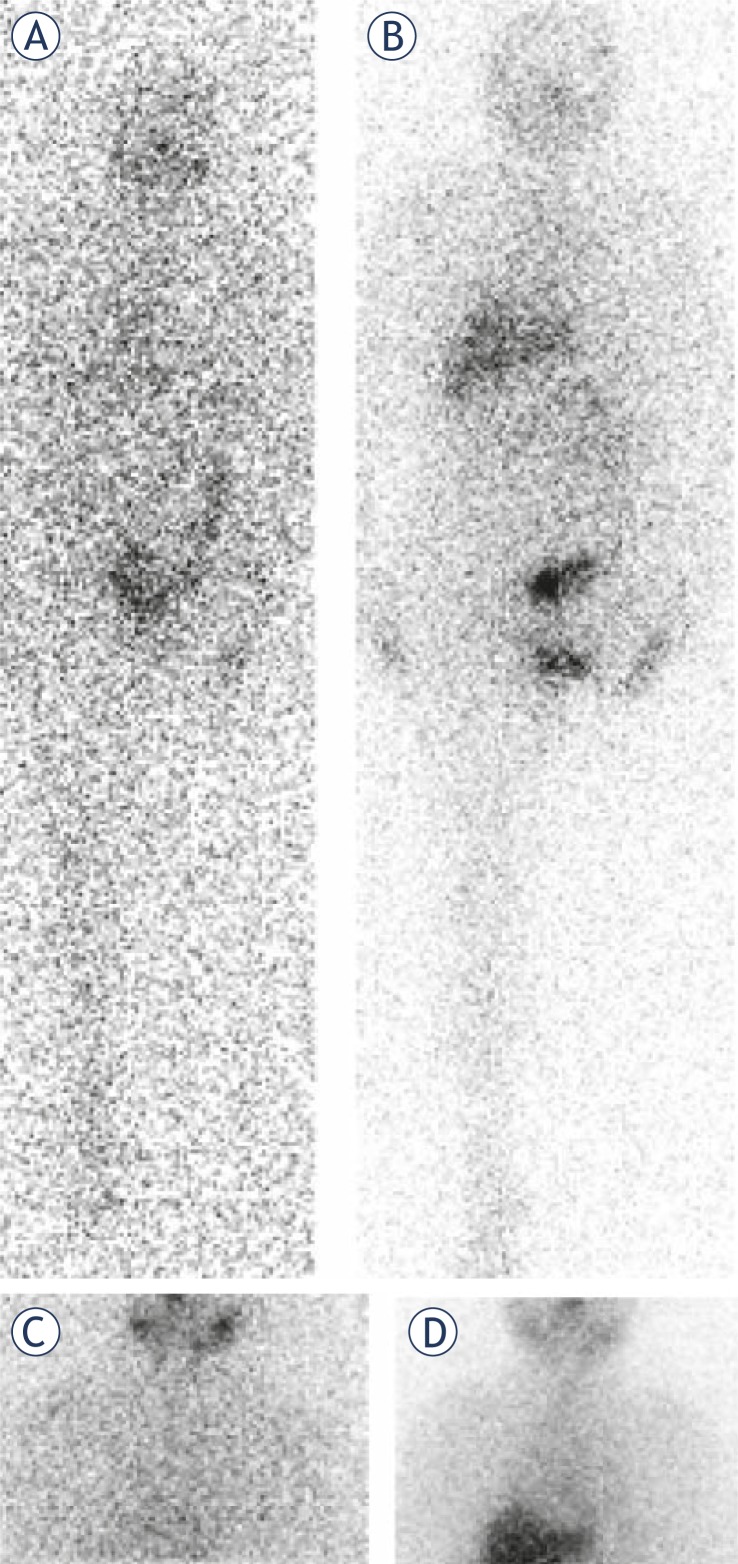
Radioiodine scans in the patient who underwent therapy with 7400 MBq (200 mCi) of ^131^I. This patient had small pulmonary nodules seen on computed tomography images performed 7 months before LID. A diffuse radioiodine uptake is seen in liver. Focal accumulation of ^131^I was not visible. Prior to LID, this patient had urinary iodine concentration of 169.6 μg/L, and post-LID value was 89.0 μg/L. Anterior whole body scans (A) prior to LID and (B) post LID; static scans of the neck and the thorax region (C) prior to LID and (D) post LID.

The patient with small pulmonary nodules seen on computed tomography images had a diffuse increased ^131^I uptake in liver after the radioiodine therapy with 7400 MBq. There was no focal or diffuse accumulation of radioiodine seen in lungs. This patient was mild iodine deficient after LID and, therefore, was suboptimal prepared for the radioiodine administration. His serum Tg level before LID and empiric radioiodine therapy was 24.9 μg/L (Figure 3).

## Discussion

In our study patients following a self-managed two-week LID significantly reduced their total iodine body content by 50%. Iodine deficient status accomplished 88% (n=14) of patients (82% of them achieved mild iodine deficiency, and 6% patients had gained moderate iodine deficient state). Two patients (12%) had the iodine sufficient status (>100 μg/L) even after the two-week LID preparation. According to Park *et al.*^4^, 78% of patients were able to achieve moderate iodine deficiency (>50 μg/L) after two-week LID. Tomoda *et al.*^6^ showed that 70% of patients reduced their urinary iodine concentration to less than 100 μg/L after two-week LID, and 35% patients were moderate iodine deficient. Possible reasons for discrepancy among data could be different regulations among countries regarding food supplementation with iodine and various dietary habits. In accordance with the legislative, all salt on the Croatian market must be iodized with 15-23 mg KI/kg NaCl.^13^ Thus, when a discontinuance of dietary iodine intake is recommended, patients have no choice but to eat unsalted food.

In our study, all fourteen patients who received ^131^I in diagnostic purposes had negative radioiodine post-LID whole body scans. All of them were mild iodine deficient or even had sufficient iodine status after two week of LID. Diffuse lung lesions in five patients, seen on computed tomography images, were small, up to 5 mm in diameter, and could be too small to be evident on radioiodine scans. Bone lesions in one patient, seen on computed tomography images, were also tiny, probably too small to be visualized on radioiodine scans. A neck lymph node metastasis in the patient who refuses the surgical treatment contains cystic tissue and, therefore, could not be visible on ^131^I scans. Post-therapy scans showed the increased diffuse radioiodine uptake in liver in one patient and focal ^131^I accumulation in the neck region in another one.

There are different data regarding to significance of the diffuse hepatic uptake seen on ^131^I whole body scans. Chung *et al*.^14^ reported the correlation between the diffuse hepatic radioiodine uptake and the thyroid remnant or metastases. Other authors did not find any connection between the thyroid remnant or recurrence and the diffuse hepatic ^131^I uptake.^15^,^16^ Omür *et al.*^16^ also find a positive correlation between the diffuse ^131^I uptake in liver and administrated ^131^I activity, increased levels of serum hepatic enzymes (AST, ALT) and hepatosteatosis.

Our patient received radioiodine therapy with 7400 MBq and the ^131^I activity was determined on an empirical basis, what is frequent at clinical praxis.^17^ The patient had hepatosteatosis and two hepatic hypovascular lesions, 7 and 10 mm in diameter, but normal serum AST and ALT levels. Due to the diffuse uptake in lungs seen on the post-ablation ^131^I whole body scan, he underwent the radioiodine therapy with 5550 MBq 10 years ago. All diagnostic radioiodine scans afterward were negative with gradually rising serum Tg levels. Hepatosteatosis and high administered activity remained only possible explanations for our patient’s radioiodine liver uptake.

Beside the ablation therapy with 1480 MBq of ^131^I five years ago and three negative radioiodine diagnostic scans afterwards, the patient who received 3700 MBq of radioiodine after LID did not receive any other ^131^I therapy. This patient achieved targeted moderate iodine deficiency, and positive finding in the neck region could be a result of thyroid remnant ^131^I avidity after two-week LID. In the further follow-up, no other morphological findings suggestive of thyroid cancer recurrence were found. Nevertheless, sensitivity of radioiodine whole body scans for detecting radioiodine avid tissue might be also improved following the administration of a high ^131^I activity.

To the best of our knowledge, there are no studies in literature related to the impact of a LID on the diagnostic or the therapeutic ^131^I administration in patients with elevated serum Tg levels and negative ^131^I whole body scans. Studies demonstrating the efficacy of a LID on postsurgical ^131^I ablation therapy are contradictory. When using the criteria of no visible uptake in the neck region and negative Tg level, Pluijmen *et al*.^5^ found a significantly higher ablation rate in patients performing a two-week LID compared to the control group (65% *vs.* 48%). On the other hand, Morris *et al.*^18^ showed no significant difference of ablation rate between two-week LID patients and those performing a regular diet (68.2% *vs.* 62.0%).

Apart from iodine contamination, radioiodine scan can be negative due to the dedifferentiation of tumour which still can produce Tg, but lost its ability to accumulate iodine, microscopic metastases which are too small to be visualized, and mutation of NIS (potassium/iodine symporter) in thyroid/ tumour cells.^19^ All these facts might be responsible for ^131^I negative scans in our patients, in spite of relatively effective LID. A rather small number of the patients and the comparison of diagnostic and therapeutic scans in two patients are clear limitations of our study. Nevertheless, encouraged with the achieved reduction of total body iodine content after LID we decided to continue with a more rigorous diet, trying, as much as we can, to exclude the influence of stable iodine on the ^131^I uptake.

## Conclusions

Our study demonstrated that two-week LID in patients with DTC was an effective way to reduce total body iodine content. Non iodized salt availability, a more stringent dietary protocol and a longer iodine restriction period are probably necessary to achieve the targeted moderate iodine deficient state in patients preparing for the ^131^I administration.

## Figures and Tables

**TABLE 1:** Epidemiological and clinical features of patients

	**Patients (N=16)**
Age (yr)	
Median	55
Range	43 – 69
Sex	
Female	11 (69%)
Male	5 (31%)
Histological type	
Papillary	10 (63%)
Follicular	6 (37%)
Peak TSH (mU/L) (Patients N=12)	
Median	72.3
Range	46.5 – 99.7
TSH> 100 mU/L (Patients N=4)	
Serum Tg concentration (μg/L)	
Median	5.0
Range	2.5 – 55.9
Administrated activity of ^131^I (MBq)	185 (diagnostic)
	3700, 7400 (therapy)
Range	185 – 7400

**TABLE 2:** Low iodine diet – recommendations by University Hospital Center Zagreb

**Avoid the following food**

Iodized salt, sea salt and salty food
Many prepared and/or cured meat (ham, bacon, sausage)
All dairy products (milk, cheese, cream, sour cream, yogurt, butter, ice cream)
Egg yolk, commercial bakery products, chocolate, dried fruit, canned vegetables, beans
Sea food and sea products (fish, shellfish, crawfish, calamari, black fish, octopus, seaweeds)
Food containing red food dyes (candies, liqueurs, cock-tails)
Iodine-containing vitamins and food supplements (check the label and ingredients and discontinue completely if iodine is included)
Medications: Betadine, Rocaltrol 0.5μg (use Rocaltrol 0.25 μg instead)

**Food that is fine to eat**

Fresh fruit and vegetable (but not too much spinach and broccoli), washed well
Vegetable can be prepared with vegetable oil and no iodized salt
Fresh no cured meat from the butcher, vegetable oil, egg white
Home-made bread (without iodized salt, milk, butter or egg yolks), pasta (without egg yolks)
Sugar , honey, clear fruit juice, tea, coffee (without milk and cream)
Canned peaches, pears and pineapples

**TABLE 3:** Urine iodine concentration (μg/L)

	**Mean ± SD**	**Median**	**Range**	
Urine iodine concentration prior to low iodine diet	153.1 ± 24.8	154.0	97.1 – 192.4	p<0.001
Urine iodine concentration after low iodine diet	76.6 ± 19.0	77.2	42.5 – 110.4	
